# RHOJ-induced chemotherapy resistance through epithelial–mesenchymal transition in drug-tolerant persister cells of head and neck cancer

**DOI:** 10.1016/j.tranon.2026.102673

**Published:** 2026-01-17

**Authors:** Hang Huong Ling, Chih-Ming Huang, Ming-Shou Hsieh, Vijesh Kumar Yadav, Iat-Hang Fong, Kuang-Tai Kuo, Chi-Tai Yeh, Jo-Ting Tsai

**Affiliations:** a Graduate Institute of Clinical Medicine, College of Medicine, Taipei Medical University, Taipei City 110, Taiwan; b Division of Hemato-oncology, Department of Internal Medicine, Chang Gung Memorial Hospital, Keelung and Chang Gung University, College of Medicine, Keelung 204, Taiwan; c Department of Otolaryngology, Taitung Mackay Memorial Hospital, Taitung City 950408, Taiwan; d Department of Nursing, Tajen University, Pingtung 90741, Taiwan; e Division of Hematology and Oncology, Taipei Medical University-Shuang Ho Hospital, New Taipei City 23561, Taiwan; f Division of Thoracic Surgery, Department of Surgery, School of Medicine, College of Medicine, Taipei Medical University, Taipei 110, Taiwan; g Division of Thoracic Surgery, Department of Surgery, Taipei Medical University-Shuang Ho Hospital, New Taipei City 235, Taiwan; h Department of Radiology, School of Medicine, College of Medicine, Taipei Medical University, Taipei City 110, Taiwan; i Department of Radiation Oncology, Cancer Center, Taipei Medical University—Shuang Ho Hospital, New Taipei City 23561, Taiwan

**Keywords:** RHOJ kinase, Tumor-associated endothelial cells, Immune escape, Head and neck squamous cell carcinoma, Epithelial–mesenchymal transition

## Abstract

Background: Building on our previous identification of TXNRD1 and RHOJ as mediators of immunotherapy resistance in head and neck squamous cell carcinoma (HNSCC), this study investigates the role of RHOJ in regulating endothelial markers and signaling pathways in drug-tolerant persister (DTP) cells, as well as its contribution to immune suppression within the resistant tumor microenvironment. Methods: We examined the role of RHOJ in regulating tumor-associated macrophage polarization and enhancing M1 antitumor activity and evaluated the therapeutic potential of targeting the RHOJ/Rho kinase axis in an HNSCC DTP mouse model. Results: RHOJ expression was significantly upregulated in HNSCC DTP cells. RHOJ contributes to chemoresistance by increasing oxidative stress and initiating the DNA damage response. RNA sequencing revealed that the IPO9/EpCAM signaling pathway positively regulates RHOJ expression. Knockdown of RHOJ with shRNA suppressed HNSCC DTP cell migration and downregulated IPO9/EpCAM signaling. Gene enrichment analysis revealed high RHOJ expression in tumor-associated endothelial cells. Treatment with the RHOJ-dependent F-actin inhibitor Latrunculin B (HY-101,848) impaired actin polymerization and increased the chemosensitivity of HNSCC DTP cells. Silencing RHOJ inhibited M2 tumor-associated macrophage activation. RHOJ overexpression promoted M2 macrophage proliferation. Conclusion: RHOJ plays a critical role in modulating immunosuppressive signaling in both tumor and endothelial cells. RHOJ promotes the function of tumor-associated endothelial cells and drives EMT through its interaction with the IPO9/EpCAM signaling pathway, thereby increasing cell survival and drug resistance. Additionally, RHOJ may limit immune cell infiltration into the tumor core and promote immune evasion by contributing to vascular abnormalities and impaired barrier function.

## Highlights

• RHOJ is upregulated in DTP cells, driving chemoresistance via oxidative stress, DNA damage response, and IPO9/EpCAM-mediated EMT.
• High RHOJ levels in endothelial cells and M2 macrophages promote immune evasion by impairing vascular integrity and limiting immune infiltration.
• Latrunculin B sensitizes HNSCC DTP cells to chemotherapy by disrupting RHOJ-dependent cytoskeletal dynamics.
• RHOJ knockdown inhibits M2 polarization and boosts M1 antitumor activity, highlighting the RHOJ/Rho kinase axis as a target to enhance immunotherapy in resistant HNSCC.

## Graphical abstract

**Figure:**
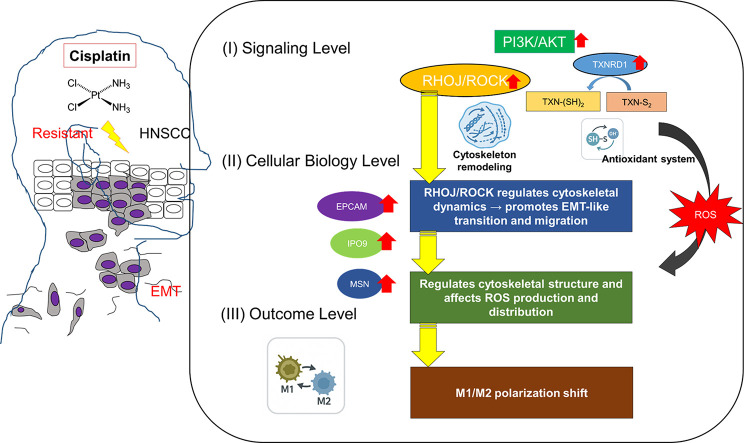

## Background

Head and neck squamous cell carcinoma (HNSCC) is a highly aggressive malignancy characterized by a high treatment failure rate, primarily resulting from drug resistance and tumor metastasis [1]. Despite advances in surgery, radiotherapy, and chemotherapy, the 5-year survival rate for patients with HNSCC is low, underscoring the urgent need for innovative therapeutic strategies. Epithelial–mesenchymal transition (EMT) has been identified as a major contributor to treatment resistance and poor prognosis. EMT is a process of cellular plasticity during which epithelial cells acquire mesenchymal characteristics, increasing their migratory, invasive, and drug-resistant capabilities [2,3]. This phenomenon facilitates both metastasis and immune evasion by modulating the tumor immune microenvironment (TME) [4,5]. Our previous study on the therapeutic potential of thioredoxin reductase 1 (TXNRD1) in promoting ferroptosis as a mechanism for immunotherapy in HNSCC revealed a complex interplay between TXNRD1 and RHOJ [6]. RHOJ is a member of the GTPase family that regulates key signaling pathways, including PI3K/AKT and NF-κB, by modulating lipid peroxidation products, influencing cellular tolerance to ferroptosis. The effects of RHOJ extend beyond ferroptosis [7,8]. RHOJ has an essential role in cytoskeletal remodeling, cellular survival, and vascular abnormalities [9].

RHOJ in endothelial cells plays a pivotal role in angiogenesis and immune evasion. RHOJ promotes vascular abnormalities and barrier function, restricting immune cell infiltration into the tumor core [10]. This activity boosts immune evasion, particularly by limiting T cell access to the tumor. RHOJ also affects cytoskeletal remodeling and regulates the production and distribution of reactive oxygen species, promoting tumor cell survival under therapeutic stress [11]. As a critical mediator of immune evasion within the TME, RHOJ modulates immunosuppressive signals, including PD-L1 expression, and inhibits immune cell infiltration [12]. Preliminary evidence suggests high RHOJ activity correlates with reduced responsiveness to PD-1/PD-L1 inhibitors, highlighting the role of RHOJ in dampening antitumor immune responses [13]. RHOJ also affects macrophage polarization by promoting M2-type tumor-associated macrophages, which support tumor growth and suppress immune responses [14]. Interventions targeting RHOJ can reprogram tumor-associated macrophages toward the proinflammatory, antitumor M1 phenotype to overcome immune suppression and improve immunotherapy outcomes. Our previously studies suggest RHOJ downregulation inhibits migration and angiogenesis in drug-tolerant persister (DTP) cells of HNSCC, a subpopulation of cells capable of surviving chemotherapy and radiotherapy [6]. Conversely, RHOJ overexpression increases the expression of Importin-9 (IPO9), EpCAM, and moesin (signaling molecules associated with EMT and tumor progression). RNA sequencing data indicate that the IPO9/EpCAM signaling pathway positively regulates RHOJ expression [15]. Additionally, the actin polymerization inhibitor Latrunculin B (HY-101,848) sensitizes DTP cells to chemotherapy through a RHOJ-dependent mechanism. The involvement of RHOJ in EMT and drug resistance underscores its promise as a therapeutic target in HNSCC. RHOJ interacts with key signaling pathways, including TGF-β, Wnt, and YAP/TAZ, to strengthen cellular survival mechanisms, contributing to treatment resistance [16,17].

By targeting RHOJ-associated cytoskeletal signaling with compounds such as Latrunculin B, it may be possible to disrupt adaptive stress-response pathways and attenuate drug tolerance. Although no selective RHOJ inhibitors are currently available, our findings suggest that therapeutically modulating the RHOJ/Rho-kinase axis could sensitize tumors to treatment. Building on this concept, we propose—as a future translational direction rather than an experiment performed in this study—that combining RHOJ-targeted strategies with immune checkpoint blockade may offer synergistic benefits by simultaneously dismantling intrinsic resistance mechanisms and alleviating immunosuppressive remodeling within the tumor microenvironment. Our data support a model in which RHOJ promotes DTP cell survival and contributes to immune exclusion partly through endothelial and stromal reprogramming. This work expands on our previous findings implicating TXNRD1–RHOJ signaling in immunotherapy resistance and provides new mechanistic insights into how RHOJ shapes tumor progression, drug tolerance, and immune escape. Targeting the RHOJ/Rho-kinase axis thus represents a promising avenue for developing next-generation therapeutic strategies in resistant HNSCC.

## Methods

### Patient selection and clinical specimen collection

This study included tissue specimens collected from 60 patients with clinically confirmed cisplatin-resistant head and neck squamous cell carcinoma (HNSCC) who underwent surgical resection at Chang Gung Memorial Hospital (CGMH), Keelung, Taiwan. All patients were treated between January 2015 and July 2020. All procedures involving human participants were reviewed and approved by the Institutional Review Board of Chang Gung Memorial Hospital (IRB No. 202301566A3 & IRB No. 202402023B0). Cisplatin resistance was defined based on histopathological confirmation of disease progression or recurrence following standard cisplatin-based chemoradiotherapy. The study cohort comprised 45 male and 15 female patients, ranging in age from 30 to 75 years (median age: 52 years). Before treatment, all patients underwent comprehensive clinical evaluations, including detailed medical history, physical examination, barium swallow radiography, upper gastrointestinal endoscopy, and chest and abdominal computed tomography (CT) imaging. All therapeutic interventions followed institutional clinical protocols aligned with the National Comprehensive Cancer Network (NCCN) guidelines. Tissue samples were obtained during primary or salvage surgery, immediately preserved in formalin, and subsequently embedded in paraffin. Archived tumor tissues were retrieved from the CGMH Biobank for retrospective analysis. Tissue microarrays (TMAs) were constructed using representative tumor cores from all 60 patients with recurrent tumors. Immunohistochemical (IHC) staining was performed to evaluate RHOJ expression using a primary anti-RHOJ antibody (1:200 dilution, ab317699; Abcam, Waltham, MA, USA). An isotype-matched mouse IgG antibody served as the negative control. IHC staining procedures followed standard protocols, and the results were independently reviewed by two board-certified pathologists who were blinded to clinical data. RHOJ expression was semi-quantitatively assessed using the Quick Score (Q-score) method, calculated as *Q* = *P* × *I*, where P denotes the percentage of positively stained tumor cells (0–100 %), and I represents staining intensity (0 = none, 1 = weak, 2 = moderate, 3 = strong), yielding a total score ranging from 0 to 300. All study procedures were approved by the Institutional Review Board of Chang Gung Memorial Hospital (approval number: 202301566A3) and conducted in accordance with the ethical principles of the Declaration of Helsinki and the Human Subjects Research Act in Taiwan. Written informed consent was obtained from all participants prior to specimen collection. To complement the clinical data, transcriptomic analyses were conducted using publicly available datasets from The Cancer Genome Atlas (TCGA) and the Gene Expression Omnibus (GEO, https://www.ncbi.nlm.nih.gov/geo/). The Expression Project for Oncology (ExpO) database was also employed to integrate gene expression profiles with longitudinal clinical data. These datasets were used to identify upstream and downstream regulatory networks associated with RHOJ expression, as well as potential diagnostic, prognostic, and therapeutic biomarkers in HNSCC.

### Tissue microarray (TMA) construction

Formalin-fixed, paraffin-embedded (FFPE) tumor blocks were selected from 60 patients diagnosed with cisplatin-resistant HNSCC. Representative tumor regions were identified on hematoxylin and eosin (H&E)-stained slides by two independent pathologists. Using a tissue microarrayer (Beecher Instruments, Silver Spring, MD, USA), 1.5-mm diameter cores were extracted in triplicate from each donor block and re-embedded into recipient paraffin blocks to construct TMAs. Serial 4-μm sections were cut and mounted on silane-coated slides for immunohistochemical staining.

### Immunohistochemistry (IHC) protocol

TMA sections were deparaffinized in xylene and rehydrated through a graded ethanol series. Antigen retrieval was performed by boiling slides in 10 mM sodium citrate buffer (pH 6.0) for 20 min using a pressure cooker. Endogenous peroxidase activity was quenched with 3 % hydrogen peroxide for 10 min at room temperature. Sections were blocked with 5 % normal goat serum for 30 min and then incubated overnight at 4 °C with anti-RHOJ primary antibody (1:200 dilution, ab317699; Abcam, Waltham, MA, USA). After rinsing with PBS, sections were incubated with HRP-conjugated anti-mouse secondary antibody (1:2000 dilution, ab97051; Abcam) for 1 hour at room temperature. Signal development was achieved using diaminobenzidine (DAB) substrate, followed by counterstaining with hematoxylin. Staining intensity and distribution were independently evaluated by two board-certified pathologists using the Quick Score (Q-score) method: *Q* = *P* × *I*, where P is the percentage of positively stained tumor cells (0–100 %) and I is the staining intensity (0 = none, 1 = weak, 2 = moderate, 3 = strong). Final scores ranged from 0 to 300.

### Bioinformatics and transcriptomic analysis

To investigate the regulatory networks and clinical relevance of RHOJ expression in head and neck squamous cell carcinoma (HNSCC), bulk RNA-sequencing datasets were obtained from The Cancer Genome Atlas (TCGA-HNSC) and the Gene Expression Omnibus (GEO; dataset IDs: GSE9844 and GSE6791). Clinical metadata for each sample were retrieved from the same data portals. Raw RNA-seq data were normalized and log₂-transformed (TPM) using the TCGA web interface and UCSC Xena browser to ensure comparability across samples. Differential gene expression and correlation analyses were conducted using the GEPIA2 platform (http://gepia2.cancer-pku.cn/, accessed on May 1, 2025), including Kaplan–Meier survival curves and associations with immune checkpoint markers. Gene Set Enrichment Analysis (GSEA) was performed to identify biological pathways and functional gene sets enriched in tumors with high versus low RHOJ expression. Expression profiles were ranked based on the signal-to-noise ratio between high-RHOJ and low-RHOJ groups, defined by the upper and lower quartiles of RHOJ expression across TCGA-HNSC samples. GSEA was conducted using the GSEA v4.3.2 software (Broad Institute, Cambridge, MA, USA) with 1000 permutations and default parameters. The curated gene sets from the Molecular Signatures Database (MSigDB), including the Hallmark, KEGG, and Reactome collections (v7.5.1), were used as references. Enrichment scores were calculated to determine the degree to which a gene set was overrepresented at the top or bottom of the ranked list, and the normalized enrichment score (NES) along with false discovery rate (FDR) q-values were used to assess significance. Pathways with an FDR < 0.25 and nominal p-value < 0.05 were considered significantly enriched.

### HNSCC cell lines, drug treatment, and derivation of drug-tolerant persister (DTP) cells

Cisplatin (purity ≥99.7 %, CAS No. 15,663–27–1) was purchased from Sigma-Aldrich (St. Louis, MO, USA). Two human head and neck squamous cell carcinoma (HNSCC) cell lines—HSC-3 and SCC-9—were obtained from Merck (USA). Cells were seeded in duplicate into 24-well plates and cultured under standard conditions until reaching approximately 70 % confluency prior to drug treatment. To induce drug-tolerant persister (DTP) cells, HSC-3 and SCC-9 cells were continuously exposed to 5 μM cisplatin for at least 9 days. The cisplatin-containing medium was replenished every 3 days to maintain consistent drug pressure. Following this treatment period, cisplatin was withdrawn, and surviving cells were allowed to recover and proliferate in drug-free medium for an additional 28 days. The drug-free medium was refreshed every 2 days throughout this recovery phase.This two-phase selection protocol enabled the enrichment and expansion of both DTP and regrown cell populations, which were subsequently used for molecular, phenotypic, and functional analyses.

### Immunofluorescence staining of CD163 in patient-derived M2 macrophages

To assess CD163 expression in M2-polarized macrophages derived from head and neck squamous cell carcinoma (HNSCC) patients, immunofluorescence staining was performed on in vitro–polarized macrophages. Fresh tumor tissues were obtained immediately after surgical resection from HNSCC patients and processed without delay. Tissues were mechanically minced and enzymatically digested using a Tumor Dissociation Kit (Miltenyi Biotec, Germany) to generate single-cell suspensions.CD14⁺ monocytes were isolated using CD14 MicroBeads (Miltenyi Biotec) and cultured in RPMI-1640 medium supplemented with 10 % fetal bovine serum, 50 ng/mL macrophage colony-stimulating factor (M-CSF), and 20 ng/mL interleukin-4 (IL-4) for 6 days to induce M2 macrophage polarization. On day 6, cells were seeded onto poly-l-lysine–coated coverslips and fixed with 4 % paraformaldehyde for 15 min at room temperature. Following fixation, cells were washed with phosphate-buffered saline (PBS), permeabilized with 0.1 % Triton X-100 for 10 min and blocked with 5 % bovine serum albumin (BSA) for 1 hour.

Cells were then incubated overnight at 4 °C with a primary antibody targeting human CD163 (rabbit anti-CD163, 1:200 dilution; Abcam, ab182422). The following day, slides were washed and incubated with a fluorescently labeled secondary antibody (goat anti-rabbit Alexa Fluor 594, 1:500 dilution; Invitrogen) for 1 hour at room temperature in the dark. Nuclear counterstaining was performed using DAPI (1 μg/mL), and coverslips were mounted using antifade mounting medium. Immunofluorescence images were acquired using a confocal laser scanning microscope (Leica TCS SP8 or Zeiss LSM 880). Quantification of fluorescence intensity and the percentage of CD163⁺ cells was performed using ImageJ software to validate M2 macrophage identity and marker expression.

### RHOJ knockdown in tumor-associated endothelial cells using shRNA

To evaluate the functional role of RHOJ in tumor-associated endothelial cells (TECs), short hairpin RNA (shRNA)-mediated knockdown was employed to stably suppress RHOJ expression in TECs isolated from primary HNSCC tumor tissues. Lentiviral transduction with RHOJ-targeting shRNA constructs was performed according to standard protocols, and stable knockdown was confirmed via qRT-PCR and Western blot analysis. RHOJ-silenced TECs were subsequently cocultured with drug-tolerant persister (DTP) HNSCC cells to simulate the tumor microenvironment (TME) in vitro. To assess the impact of RHOJ depletion on endothelial cell identity and signaling, the expression of key endothelial and EMT-associated markers—including IPO9, MSN (moesin), EpCAM, TGFB1, CTNNB1, VEGFR2, and CD31—was quantified using real-time PCR. This coculture model enabled the functional interrogation of RHOJ-mediated signaling between TECs and DTP cells in the context of drug resistance and tumor progression.

### Orthotopic HNSCC mouse model and drug treatment

To establish an orthotopic head and neck squamous cell carcinoma (HNSCC) model, 2 × 10⁶ drug-tolerant persister (DTP) cells were injected into the lateral tongue of 6–8-week-old NOD/SCID mice (IACUC approval number: LAC2023–0302). Tumor formation was confirmed by physical examination and caliper measurements two weeks post-injection. Mice were then randomized into four treatment groups (*n* = 15 per group): (1) vehicle control; (2) F-actin polymerization inhibitor (Latrunculin B, 2 mg/kg, intraperitoneally); (3) cisplatin (10 mg/kg, intraperitoneally); and (4) combination therapy with both agents at the indicated doses. Treatments were administered according to their respective schedules for 4 weeks. At the study endpoint, mice were euthanized, and tongue tumors were excised and weighed. Tumor tissues were fixed in formalin, embedded in paraffin, and sectioned for immunohistochemical (IHC) analysis. IHC was performed to evaluate proliferation (Ki-67), apoptosis (cleaved caspase-3), RHOJ and IPO9. Quantitative comparisons among groups were used to assess the efficacy and mechanistic impact of F-actin inhibition and chemotherapy on drug-resistant HNSCC tumors.

### Statistical analysis

All experiments were performed in triplicate and independently repeated at least three times. Data are presented as mean ± standard deviation (SD). For comparisons between two groups, statistical significance was assessed using an unpaired two-tailed Student’s *t*-test. For analyses involving multiple groups, one-way or two-way analysis of variance (ANOVA) was applied, followed by Tukey’s or Bonferroni’s post hoc tests, as appropriate. Kaplan–Meier survival curves were analyzed using the log-rank (Mantel–Cox) test. Pearson’s correlation coefficient was used to evaluate linear associations between variables. All statistical analyses were conducted using GraphPad Prism version 9.0 (GraphPad Software, San Diego, CA, USA). A p-value < 0.05 was considered statistically significant.

## Results

### Relationship between high RHOJ expression in cisplatin-resistant HNSCC and M2 polarization, cytoskeletal remodeling, and stress-adaptation pathways

**Fig. 1:**
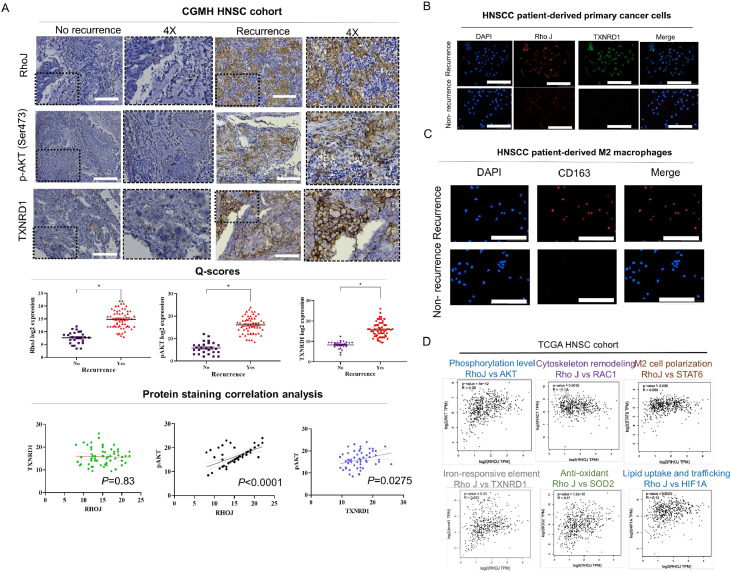
Association of High RHOJ Expression in Cisplatin-Resistant HNSCC with M2 Polarization, Cytoskeletal Remodeling, and Stress Adaptation. (A) IHC staining revealed significantly elevated RHOJ expression in recurrent, cisplatin-resistant HNSCC tissues compared with nonrecurrent, cisplatin-sensitive samples. Quantification using the Quick score method revealed increased staining in tumor cell membranes and endothelial regions (p < 0.001). Parallel pAKT (Ser473) IHC staining in the same cohort showed that pAKT Q-scores positively correlated with RHOJ expression, supporting a link between RHOJ upregulation and AKT pathway activation in resistant tumors. (B–C) immunofluorescence analysis demonstrated colocalization of RHOJ with CD31⁺ endothelial cells and CD206⁺ M2 macrophages, suggesting its role in vascular remodeling and immune suppression. (D) Transcriptomic correlation analysis revealed the positive association of RHOJ expression with AKT (phosphorylation signaling), RAC1 (cytoskeletal remodeling), STAT6 (M2 polarization), and SOD2 (oxidative stress response).

Table 1 shows clinicopathological characteristics and stratification of patients included in this study, along with corresponding RHOJ expression levels. The table summarizes detailed demographic and clinical variables. RHOJ IHC Q-scores are presented across these strata to illustrate expression differences in resistant versus sensitive disease. To avoid confounding effects arising from the distinct biology of HPV-driven tumors, our mechanistic analyses and tissue-based evaluations in this study were focused on HPV-negative (non–virus-induced) HNSCC, which represents the predominant subtype in our cohort. Therefore, the observed RHOJ-associated chemoresistance and stress-adaptation phenotypes primarily reflect the biology of HPV-negative HNSCC. This table provides the clinical context supporting the relevance of RHOJ dysregulation in nonviral, treatment-resistant HNSCC. A patient inclusion flowchart is provided in **Supplementary Figure S1**, summarizing screening procedures, eligibility criteria, exclusion steps, and the final allocation of cases into cisplatin-sensitive and cisplatin-resistant subgroups in accordance with the clinical stratification variables listed in Table 1. IHC analysis revealed that RHOJ expression was higher in tumor tissues from patients with recurrent, cisplatin-resistant HNSCC than in those from patients with nonrecurrent, cisplatin-sensitive HNSCC. The Q-score analysis showed significantly elevated RHOJ expression within resistant tumors (*p* < 0.001), with RHOJ predominantly localized to the plasma membrane of tumor cells and tumor-associated endothelial cells (TECs) (Fig. 1**A**). To further connect RHOJ to downstream survival signaling, we additionally performed pAKT (Ser473) IHC staining and Q-score quantification in the same patient cohort. The analysis demonstrated a positive correlation between RHOJ and pAKT levels, supporting a functional link between RHOJ overexpression and AKT pathway activation in resistant tumors. This spatial pattern suggests that RHOJ participates in both tumor cell plasticity and vascular remodeling. Immunofluorescence costaining further validated RHOJ localization within the TME; RHOJ colocalized with the endothelial marker CD31 and the M2 macrophage marker CD206, supporting its involvement in angiogenesis and immunosuppressive macrophage recruitment/polarization (Fig. 1**B–C**). To elucidate the functional relevance of RHOJ overexpression, we performed correlation analyses of both transcriptomic data and protein-level IHC scores. Consistent with its role in survival signaling, RHOJ expression showed a positive correlation with pAKT (Ser473) IHC Q-scores in our patient cohort, indicating that RHOJ activation converges on AKT-mediated stress-response pathways. In transcriptomic datasets, RHOJ expression was positively correlated with RAC1 (*p* = 0.0015), a central actin-cytoskeleton regulator; STAT6 (*p* = 0.036), a key mediator of M2 polarization; and SOD2 (*p* < 1e−10), a mitochondrial antioxidant enzyme. We also observed weaker but consistent associations of RHOJ with hypoxia-driven metabolic regulators such as HIF1A and redox-regulatory genes including TXNRD1, supporting a broader role for RHOJ in metabolic and oxidative-stress adaptation (Fig. 1**D**). Importantly, TXNRD1 IHC Q-scores also correlated with pAKT, situating both TXNRD1 and RHOJ upstream of AKT activation and providing a functional, protein-level bridge despite the absence of transcriptional correlation in TCGA. Together, these findings highlight RHOJ as a central regulatory node linking chemoresistance, cytoskeletal remodeling, metabolic reprogramming, and immune evasion, particularly within DTP-like tumor populations in HNSCC.

**Table 1:** The clinical characteristics of RHOJ expression levels, and treatment responses.

Clinicopathological VariablesT4		RHOJ			
	No.	High expression	Low expression	x^2^	p-value
Age, years					
<65	30	15	15	1.08	0.29
>65	30	19	11		
Gender					
Male	45	29	16	4.43	0.03
Female	15	5	10		
Differentiation					
Well/Moderately	20	5	15	17.06	0.0001
Poor	40	32	8		
Lymph node metastasis					
Dis-metastasis	20	4	16	20	0.0001
metastasis	40	32	8		
Primary Stage					
M0	25	12	13	0.068	0.79
M1	35	18	17		
T category					
T1	7	4	3	4.68	0.197
T2	8	5	3		
T3	25	20	5		
T4	20	18	2		
N category					
N0	20	4	16	25.93	0.00001
N1	10	7	3		
N2	12	10	2		
N3	18	17	1		
HPV/p16 status					
Positive	12	5	7	3.97	0.04617
Negative	48	36	14		
Treatment modality					
Surgery only	15	8	7	8.88	0.0028
Surgery + adjuvant CRT	45	40	5		
Cisplatin regimen					
Weekly	25	15	10	0.85	0.35
3-week high dose	35	25	10		
Specimen source					
Surgical tissue	15	8	7	8.88	0.0028
post-CRT biopsy	45	40	5

### Molecular and cellular adaptations in DTP SCC9-p cells

**Fig. 2:**
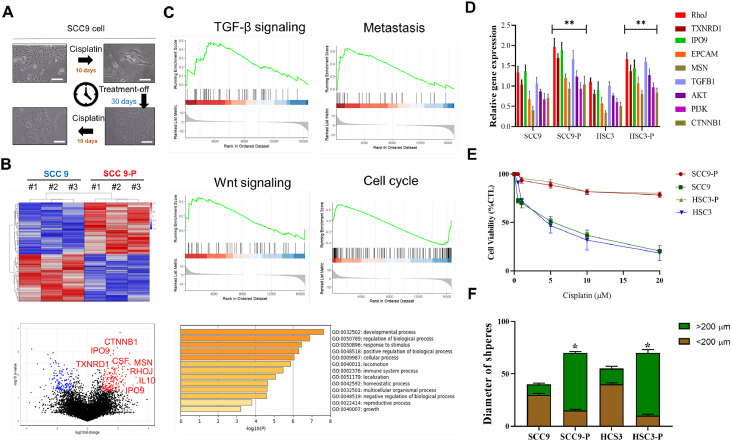
Molecular and Cellular Adaptations in DTP SCC9-P Cells. (A) Morphological comparison of SCC9 and SCC9-P cell cultures analyzed after 10 and 30 days. (B) Heatmap of differential gene expression profiles between SCC9 and SCC9-P cells and volcano plot illustrating significant upregulation of RHOJ, IPO9, and IL10 in SCC9-P cells. These genes are associated with cytoskeletal remodeling, immune regulation, and drug resistance. (C) Gene barcode map analysis and bar graph summary analysis. (D) Bar plot demonstrating differential expression of genes related to EMT, immune regulation, and reactive oxygen species metabolism between SCC9 and SCC9-P cells. Immune evasion genes (RHOJ, IL10) and cytoskeletal dynamics genes (MSN, IPO9) were significantly upregulated in SCC9-P cells. (E) Cell viability assays revealed significantly higher resistance to chemotherapy of SCC9-P than SCC9 cells. (F) Tumor sphere formation assay comparing SCC9 and SCC9-P cells. SCC9-P cells exhibited significantly larger tumorsphere diameter, underscoring their greater cancer stemness, cytoskeletal changes, and survival potential after drug resistance.

SCC9 and SCC9-P cells were cultured for 30 days. The morphology of these cells was examined under a microscope at days 10 and 30. The SCC9-P cells, a DTP cell variety, exhibited significant morphological changes, including elongated shape and increased cytoplasmic volume compared with SCC9 cells. These observations likely indicate cytoskeletal remodeling and cellular adaptations in SCC9-P cells, possibly contributing to their drug-resistant phenotype (Fig. 2**A**). RNA sequencing heatmap analysis revealed differences in the gene expression profiles of SCC9 and SCC9-P cells. Genes involved in ferroptosis resistance (TXNRD1, RHOJ) and immune modulation (IL10, CTNNB1) were significantly upregulated in SCC9-P cells, whereas metabolic and apoptotic pathway genes were downregulated in these cells (Fig. 2**B**). These molecular changes support drug resistance and immune evasion in SCC9-P cells. The figure illustrates the significant upregulation of key genes, including RHOJ, IPO9, and IL10, in SCC9-P cells (*p* < 0.05). These genes are implicated in cytoskeletal remodeling, immune regulation, and drug resistance, reinforcing their central role in the DTP phenotype (Fig. 2**B**). Gene barcode map analysis and bar graph summary revealed a relationship between the regulatory molecular pathways potentially associated with RHOJ in DTP cells and TGF-β signaling, metastasis, cell cycle regulation, and Wnt signaling (Fig. 2**C**). The bar plots demonstrate the differential expression of genes involved in key signaling pathways (e.g., EMT, immune regulation, and reactive oxygen species metabolism) between SCC9 and SCC9-P cells. Notably, genes associated with immune evasion (RHOJ, IL10) and cytoskeletal dynamics (MSN, IPO9) were upregulated in SCC9-P cells, highlighting their role in DTP cell adaptation and survival (Fig. 2**D**). To functionally validate the drug-resistant phenotype, viability assays were conducted on SCC9 and SCC9-P cells under chemotherapy treatment. Significantly higher survival rates were observed for SCC9-P than SCC9 cells, indicating their tolerance to therapeutic insult (Fig. 2**E**). This hallmark finding underscores the clinical relevance of DTP populations, which often remain dormant or minimally responsive during therapy and later drive relapse. Our findings confirm the drug tolerance phenotype in vitro, underscoring the necessity of strategies to eradicate SCC9-P cells or reverse their resistant state. We also investigated the capacity of SCC9 and SCC9-P cells to sustain three-dimensional growth and self-renewal (proxies for cancer stem cell properties) through a tumor sphere formation assay. SCC9-P cells exhibited notably larger and more numerous tumor spheres, correlating with their upregulated gene expression in EMT, immune regulation, and cytoskeletal remodeling pathways (Fig. 2**F**).

### RHOJ as a key regulator of tumor microenvironment remodeling in HNSCC

**Fig. 3:**
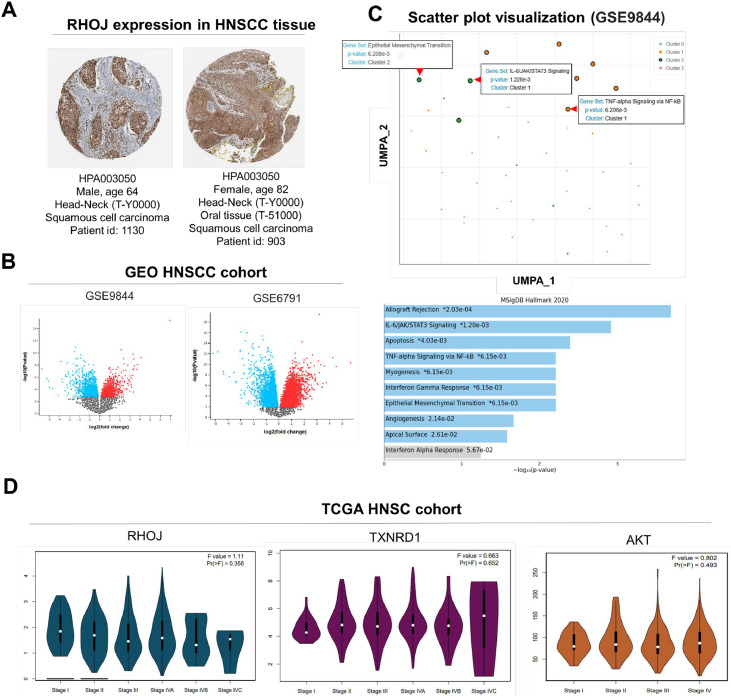
Prognostic and Molecular Role of RHOJ in HNSCC Progression and Immune Evasion. (A) IHC staining of RHOJ in HNSCC tumor tissues compared with normal tissues. Dense and widespread staining of tumor samples indicates elevated RHOJ levels in the TME. (B) Volcano plots for datasets GSE9844 and GSE6791 revealing significant upregulation of RHOJ and TXNRD1 (red dots) in tumor samples compared with normal samples (blue dots). (C) Scatter plot visualization (GSE9844) (D) Violin plots illustrating consistent RHOJ expression across all HNSCC stages, suggesting the role of RHOJ in regulating tumor progression without pronounced stage-dependent variation.

We observed significantly elevated RHOJ expression in tumor tissues compared to adjacent normal epithelium, with dense cytoplasmic and perivascular staining localized to regions of tumor–stroma interaction. RHOJ was prominently expressed in tumor-associated endothelial and stromal compartments, supporting its role in shaping an immunosuppressive and pro-tumorigenic microenvironment (Fig. 3**A**). Transcriptomic analyses from GSE9844 and GSE6791 further demonstrated increased expression of RHOJ and its associated gene TXNRD1 in tumor samples relative to normal controls, reinforcing the link between RHOJ overexpression, malignant phenotypes, and TME remodeling (Fig. 3**B**). Further analysis of molecular targets associated with the RHOJ–AKT–TXNRD1 axis in GSE9844 was performed using scatter plot–based correlation mapping (Fig. 3**C**). This approach enabled visualization of coordinated transcriptomic changes linked to cytoskeletal remodeling, redox regulation, and stress-adaptive signaling. From this analysis, we systematically identified the top ten genes most strongly correlated with RHOJ pathway activity, reflecting key effectors involved in actin dynamics, oxidative defense, and immune–microenvironmental remodeling. These genes and their statistical significance are summarized in Table 2, providing a focused molecular profile that further supports the central role of the RHOJ–AKT–TXNRD1 signaling axis in driving chemoresistance and tumor microenvironment adaptation in HNSCC. Violin plots showed that RHOJ levels remained consistently elevated across HNSCC stages, indicating that its function is not stage-dependent but rather reflects a core regulatory role in cytoskeletal remodeling, immune modulation, and stress adaptation within the TME. The absence of significant variation between early- and late-stage disease (Fig. 3**D**) suggests that RHOJ acts as a foundational driver of tumor–microenvironment interactions, rather than a stage-associated or prognostic marker.

**Table 2:** Top 10 Upregulated Genes Related to RHOJ–AKT–TXNRD1 Axis (GSE9844).

Gene	logFC	Biological Function / Pathway Link	significance
IL1B	+1.512	Pro-inflammatory cytokine; activates NF-κB & AKT	Links oxidative stress & AKT activation; EMT promoter
STAT1	+1.40	Interferon signaling; oxidative stress modulation	Upstream of TXNRD1; enhances invasion via EMT
SPARC	+1.442	ECM remodeling; tumor invasion	Works with RHOJ-driven actin/adhesion remodeling
LAMC1	+1.043	Laminin γ1; integrin → FAK/PI3K/AKT	Direct upstream activator of AKT pathway
GNB1	+1.007	GPCR G-protein subunit → PI3K/AKT	AKT pathway activation node
APP	+0.996	Regulates cytoskeleton & Rac/Rho GTPase	Functionally intersects with RHOJ-like pathways
MSN (Moesin)	+0.934	Actin cytoskeleton remodeling (ERM family)	Direct downstream effector of Rho GTPase activity
LITAF	+0.915	TNF/Inflammatory/ROS pathways	Functionally tied to TXNRD1 (redox balance)
BCAT1	+2.219	Metabolic rewiring; EMT activation	Enhances invasion & metastasis (RHOJ phenotype)
CMPK2	+2.261	IFN-inducible mitochondrial enzyme (ROS-related)	Strong oxidative-stress/TXNRD1–linked gene

### Single-Cell and molecular analysis of RHOJ in HNSCC TME

**Fig. 4:**
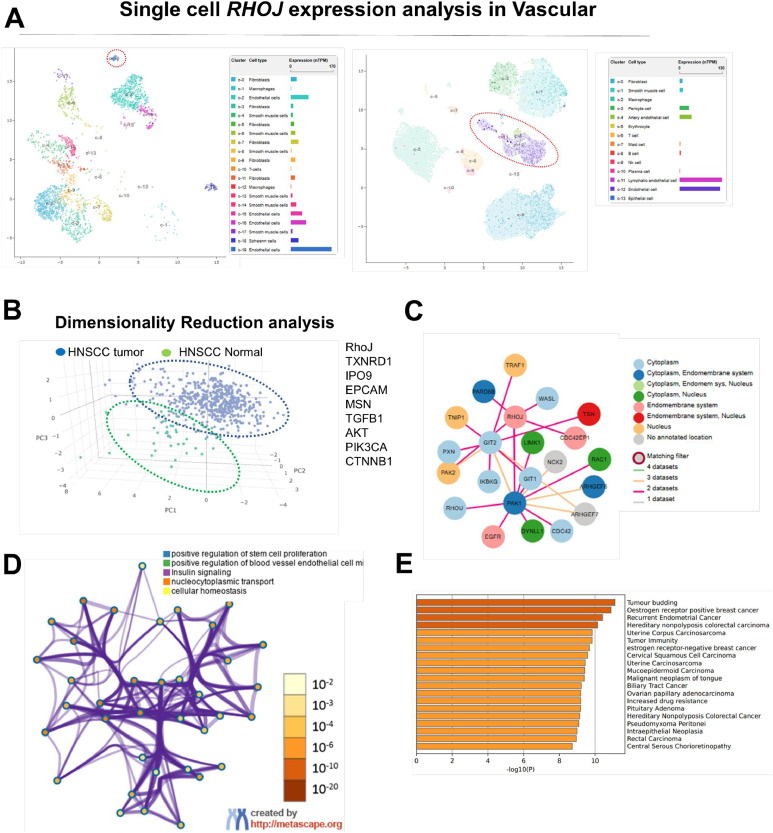
Single-Cell and Molecular Analysis of RHOJ in HNSCC TME. (A) t-SNE plot of single-cell RNA sequencing data, with distinct cell clusters observable in HNSCC TME. (B) Principal component analysis results. Tumor-specific clustering reflects RHOJ-associated transcriptomic differences in tumor progression and TME remodeling. (C) Protein–protein interaction network, in which RHOJ is a central node that interacts with key signaling regulators.

Distinct cell clusters in the HNSCC TME were observed in t-SNE plots of single-cell RNA sequencing data. High RHOJ expression was noted in cluster 10, which had high endothelial cell enrichment, suggesting the involvement of RHOJ in vascular regulation and immune evasion. Analysis of immune and stromal cell clusters revealed differential RHOJ expression across cell types, particularly in macrophages and fibroblasts (Fig. 4**A**). Principal component analysis indicated clear segregation between HNSCC tumor samples and normal samples, confirming RHOJ-associated transcriptomic differences in tumor progression. Tumor samples exhibited unique clustering, reflecting alterations in TME and RHOJ-associated pathways (Fig. 4**B**). In the protein–protein interaction network, RHOJ was identified as a central node interacting with key signaling regulators, including PAK1, LIMK1, and EGFR (Fig. 4**C**). Furthermore, we visualized the pathway and process enrichment analysis using the Metascape platform (https://metascape.org/gp/index.html#/main/step1). This analysis revealed RHOJ-related gene sets enriched in processes such as stem cell proliferation, endothelial cell migration, insulin signaling, and cellular homeostasis, further supporting its role in vascular remodeling and metabolic adaptation (Fig. 4**D**). In addition, cancer type enrichment analysis demonstrated that RHOJ and its associated gene network were significantly linked to tumor phenotypes including tumor budding, HER2-enriched and estrogen receptor-positive breast cancer, squamous cell carcinoma, and metastatic colorectal cancer (Fig. 4E). This finding implicates RHOJ in cytoskeletal remodeling, immune suppression, and cell survival pathways.

(D) Pathway and process enrichment analysis and (E) Summary of enrichment analysis in DisGeNET (https://metascape.org/gp/index.html#/main/step1, accessed on 12 May 2025.)

### Effect of RHOJ inhibition on tumor cell migration and chemosensitivity in scc9-p cells

**Fig. 5:**
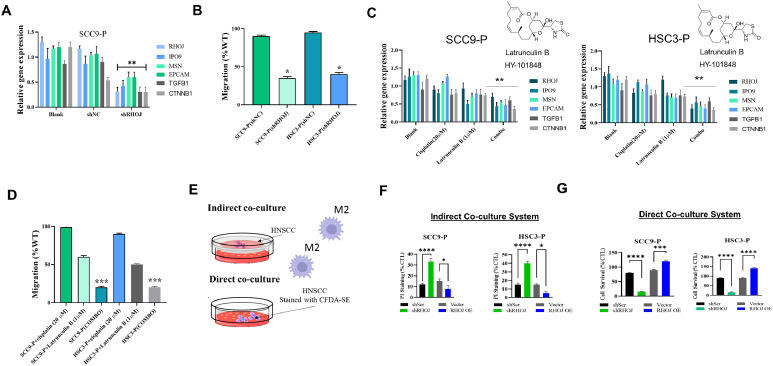
Effect of RHOJ on Tumor Cell Migration and Chemosensitivity in SCC9-P Cells. (A) shRNA-mediated knockdown of RHOJ in SCC9-P cells significantly downregulated IPO9, MSN, and EpCAM expression. (B) Migration assays revealed that RHOJ inhibition reduces the migratory capability of SCC9-P cells. (C) Treatment with F-actin inhibitor Latrunculin B (HY-101,848) disrupted actin polymerization, downregulating tumor-related network genes in SCC9-P cells. (D) Combined treatment with Latrunculin B and cisplatin increased chemosensitivity in a RHOJ-dependent manner, effectively targeting DTP cells. (E) Direct and indirect coculture system setup illustrating interaction between M2 macrophages and HNSCC cells. (F) Indirect coculture indicating that RHOJ silencing reduces SCC9-P and HSC3-P cell viability, whereas RHOJ overexpression increases cell growth. (G) Direct coculture indicating that RHOJ silencing inhibits M2 macrophage activation, whereas RHOJ overexpression significantly promotes M2 cell proliferation.

We used shRNA to inhibit RHOJ expression in SCC9-P cells to investigate the molecular regulatory role of RHOJ (Fig. 5**A**). The results indicated that RHOJ inhibition affects the expression of IPO9, MSN, EpCAM, and related genes, highlighting the involvement of RHOJ in TEC regulation and immune evasion. As displayed in Fig. 5**B**, RHOJ inhibition significantly inhibited SCC9-P cell migration, potentially through its role in EMT. Treatment with the F-actin inhibitor Latrunculin B (HY-101,848) disrupted actin polymerization, downregulating tumor-related network genes and further inhibiting cell migration (Figs. 5**C–D**). Notably, combined Latrunculin B and cisplatin (a chemotherapy drug) increased chemosensitivity in a RHOJ-dependent manner, effectively targeting DTP cells. These results suggest that RHOJ may be a promising therapeutic target for antiangiogenic and combination therapies in HNSCC. The TME supports M2 macrophages in suppressing the host immune response. To investigate the role of RHOJ in promoting M2 cell proliferation, we established a direct and an indirect coculture system of M2 macrophages and HNSCC cells (Fig. 5**E**). In the indirect coculture system, a transmembrane layer separated M2 and HNSCC cells, preventing direct interaction. RHOJ silencing reduced the viability of SCC9-P and HSC3-P cells, whereas aberrant RHOJ expression rescued and increased the growth of these HNSCC cell lines (Fig. 5**F**). Similarly, in the direct coculture system, where M2 macrophages could directly interact with HNSCC cells, RHOJ silencing inhibited M2 cell activation, whereas RHOJ overexpression promoted M2 cell activation and proliferation (Fig. 5**G**). Validation in additional HNSCC cell lines is shown in **Supplementary Figure S2**, demonstrating that the observed effects are reproducible across multiple models.

### F-actin polymerization inhibition attenuates tumor growth and drug resistance in an orthotopic HNSCC DTP mouse model

**Fig. 6:**
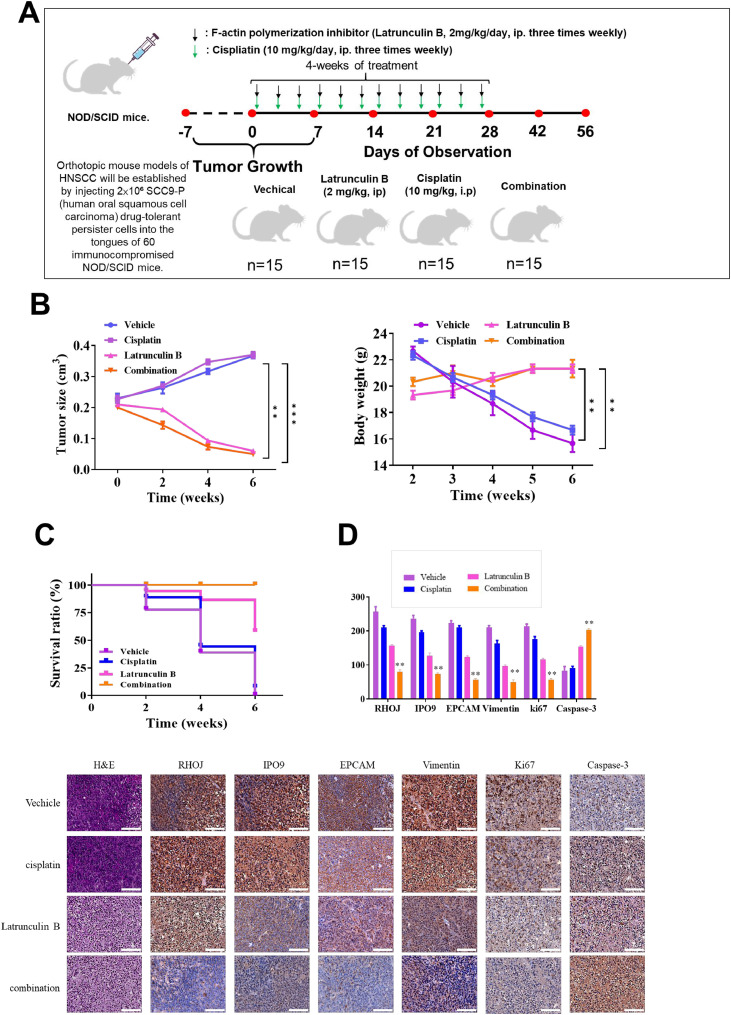
Inhibition of F-actin polymerization enhances cisplatin sensitivity and suppresses tumor growth in an orthotopic HNSCC DTP mouse model. (A) Representative tumor growth curves in NOD/SCID mice bearing orthotopic tongue tumors derived from HNSCC DTP cells. Mice were randomized into four groups: vehicle control, cisplatin (10 mg/kg, i.p.), F-actin polymerization inhibitor (2 mg/kg, i.p.), and combination therapy. (B) Combination treatment resulted in a significant reduction in tumor volume compared to monotherapies. (C) Kaplan–Meier survival analysis of mice over the 4-week treatment period. The combination group exhibited significantly prolonged survival (log-rank test, p < 0.05). (D) Representative immunohistochemistry (IHC) staining of excised tumor tissues for Ki-67 (proliferation), cleaved caspase-3 (apoptosis), IPO9 (angiogenesis), and RHOJ. Decreased Ki-67, IPO9, and RHOJ expression, and increased cleaved caspase-3 staining were observed in the combination group. Quantitative IHC scoring (Q-score) of indicated markers. Data are presented as mean ± SD (n = 15 per group). Statistical significance was determined using one-way ANOVA with Tukey’s post hoc test. p < 0.05; p < 0.01.

To evaluate the therapeutic potential of targeting drug-tolerant persister (DTP) cells in head and neck squamous cell carcinoma (HNSCC), an orthotopic xenograft mouse model was established by injecting DTP cells into the lateral tongues of immunodeficient NOD/SCID mice. Two weeks after inoculation, palpable tumors were confirmed, and mice were randomized into four treatment groups (15 animals per group): (1) vehicle control, (2) cisplatin alone (10 mg/kg, i.p.), (3) F-actin polymerization inhibitor alone (2 mg/kg, i.p.), and (4) a combination therapy group receiving both agents. The experimental design and treatment schedule are illustrated in Fig. 6**A**. Monotherapy with the F-actin inhibitor led to a moderate decrease in tumor volume compared to control animals. Notably, combination treatment with the F-actin inhibitor and cisplatin resulted in the most significant tumor suppression, reflected by markedly reduced tumor size and weight after four weeks of treatment (*p* < 0.01, Fig. 6**B**). Body weight remained stable across all groups, indicating that the treatments were well tolerated. Kaplan–Meier survival analysis revealed that mice receiving combination therapy exhibited significantly prolonged survival compared to those receiving either monotherapy (Fig. 6**C**). Histological and immunohistochemical analyses of excised tumors showed substantial downregulation of RHOJ, IPO9, and Ki-67 expression in the F-actin inhibitor and combination groups, suggesting reduced tumor proliferation and signaling activity. In parallel, levels of cleaved caspase-3 were significantly elevated, indicating enhanced apoptotic responses. RHOJ and IPO9 expression were also reduced, pointing to suppressed angiogenesis and decreased drug efflux capacity. Quantitative IHC scoring (Q-scores) supported these findings, reinforcing the role of F-actin polymerization in promoting tumor growth, chemoresistance, and immune evasion in HNSCC DTP cells. Collectively, these results demonstrate that co-targeting cytoskeletal dynamics alongside chemotherapy may provide a promising strategy to overcome therapeutic resistance in refractory HNSCC (Fig. 6**D**).

## Discussion

DTP cells are a major obstacle in the treatment of HNSCC, a malignancy widely known for its high recurrence rate and poor clinical outcomes [18]. The findings of this study implicate RHOJ, a member of the Rho GTPase family, as a central regulator of chemoresistance in HNSCC DTP cells, primarily through its effects on EMT, cytoskeletal remodeling, and TME interactions [19]. These results contribute to the growing body of evidence linking RHOJ to aggressive tumor phenotypes, highlighting its multifaceted role in sustaining therapy-resistant cell populations [20,21]. A major finding of this study is the consistent upregulation of RHOJ in DTP cells following cisplatin treatment. RHOJ has been implicated in angiogenesis and cytoskeletal reorganization in endothelial cells. Nevertheless, its role in cancer cell plasticity and chemoresistance remains unclear. Our data demonstrate that RHOJ expression increases oxidative stress tolerance and promotes DNA damage response pathways, both of which are crucial for the survival of cells exposed to genotoxic agents. These findings suggest that RHOJ may regulate stress responses, enabling DTP cells to persist under chemotherapy-induced damage. Notably, RHOJ knockdown significantly restored chemosensitivity in DTP cells, supporting the role of RHOJ as a driver of persistent drug resistance. Mechanistically, RHOJ expression was positively regulated by the IPO9/EpCAM axis. IPO9, a nuclear import receptor, controls cell cycle progression and chromatin organization [17]. EpCAM is a key epithelial marker associated with cancer stemness and metastasis. The interaction between IPO9 and EpCAM in promoting RHOJ expression highlights the convergence of nuclear transport and epithelial signaling pathways in regulating the drug-resistant phenotype [22]. When RHOJ was silenced, IPO9 and EpCAM were downregulated, suggesting the existence of a positive feedback loop that may sustain EMT-associated transcriptional programs [23].

During EMT, epithelial tumor cells acquire mesenchymal characteristics, allowing them to become more motile, invasive, and resistant to apoptosis [24,25]. In our model, RHOJ overexpression was associated with the upregulation of canonical EMT markers, including N-cadherin, vimentin, and Snail. shRNA mediated RHOJ silencing reversed this expression pattern. These findings indicate that RHOJ plays a causative role in EMT induction. The increased migratory ability of DTP cells further supports this notion, providing a mechanistic link between RHOJ activity and metastatic potential. RHOJ also plays a critical role in regulating TECs. Gene enrichment and tissue profiling revealed high RHOJ expression in endothelial compartments within the TME [10,26]. Functionally, RHOJ contributes to vascular remodeling and actin cytoskeleton dynamics, both of which are essential for angiogenesis and tumor perfusion. Treatment with the actin polymerization inhibitor Latrunculin B, which selectively impairs RHOJ-dependent F-actin assembly, significantly reduced tumor cell viability and increased sensitivity to chemotherapy [27,28]. These results suggest that RHOJ-mediated cytoskeletal remodeling both supports EMT and promotes endothelial barrier dysfunction, potentially facilitating tumor cell dissemination and limiting immune cell infiltration. The immunological dimension of RHOJ function was further elucidated through coculture experiments involving tumor-associated macrophages [29]. RHOJ silencing inhibited the polarization and proliferation of M2-like tumor-associated macrophages, and RHOJ overexpression had the opposite effect. Given that M2 tumor-associated macrophages promote immune suppression, tumor growth, and angiogenesis [30], our findings suggest RHOJ mediates immune evasion. RHOJ modulates the TME to promote M2 macrophage activation, potentially indirectly suppressing cytotoxic T cell recruitment and increasing immunosuppressive cytokine secretion, such as IL10 and TGF-β. These immunological changes, in combination with EMT and endothelial dysfunction, could create a permissive niche for DTP cell survival and expansion.

The results presented in Fig. 1 support the multifaceted role of RHOJ in promoting chemoresistance and tumor progression in HNSCC. Elevated RHOJ expression in cisplatin-resistant tumors, particularly within endothelial cells and tumor-associated macrophages, suggests that RHOJ supports the TME. The colocalization of RHOJ with CD206⁺ M2 macrophages and CD31⁺ vasculature indicates potential crosstalk between RHOJ-driven signaling and immunosuppressive or angiogenic pathways. Transcriptomic correlation analyses further revealed that RHOJ expression is linked to key regulators of cell survival (AKT), cytoskeletal dynamics (RAC1), oxidative stress adaptation (SOD2), and M2 macrophage polarization (STAT6). These findings suggest that RHOJ is a molecular integrator of tumor-intrinsic and microenvironmental stress-response programs. Therapies targeting the RHOJ axis may therefore disrupt both intrinsic resistance mechanisms and extrinsic immune modulation, offering a promising strategy in refractory HNSCC. Fig. 2 illustrates the morphological and molecular distinction of SCC9-P cells from parental SCC9 cells, underscoring how DTP cell populations deploy a suite of adaptive changes to survive chemotherapy. The molecular networks underlying these changes, which include RHOJ, IPO9, IL10, and ferroptosis regulators, can be further studied to foster the development of novel therapeutic strategies to overcome drug resistance, prevent recurrence, and improve patient outcomes in HNSCC. The increased sphere-forming ability of SCC9-P cells suggests an elevation in stem-like properties, which are frequently linked to apoptosis resistance, metabolic flexibility, and immune evasion. Cells displaying these stem-like attributes are often implicated in relapse and metastatic spread. Collectively, these observations provide preliminary evidence regarding the interconnectedness of morphological shifts, cytoskeletal reorganization, immune evasion, and ferroptosis resistance within the DTP phenotype. Fig. 3 highlights RHOJ as a pivotal player in tumor progression and immune evasion in HNSCC. Correlations with key immune and EMT-related genes further support the role of RHOJ in orchestrating TME remodeling and therapeutic resistance. Novel therapeutic strategies targeting RHOJ may therefore improve clinical outcomes for patients with HNSCC. Fig. 4 highlights the central role of RHOJ in the HNSCC TME, where it regulates endothelial function, immune evasion, and cytoskeletal dynamics. Elevated RHOJ expression in endothelial cells and macrophages reveals RHOJ is a critical driver of tumor angiogenesis and immune suppression, highlighting its potential as a therapeutic target to reshape the TME and improve treatment outcomes. Fig. 5 illustrates the critical role of RHOJ in tumor progression and immune modulation in HNSCC. RHOJ inhibition impairs tumor cell migration, disrupts actin polymerization, and sensitizes cells to chemotherapy when combined with Latrunculin B and cisplatin. Moreover, RHOJ facilitates M2-macrophage activation and their crosstalk with tumor cells, thereby enhancing immune evasion. Therapeutically, these effects are mitigated by the concomitant down-regulation of RHOJ and IPO9—critical regulators of actin dynamics and nuclear import that are essential for DTP-cell survival. Our findings in Fig. 6 demonstrate that pharmacological inhibition of F-actin assembly, when combined with cisplatin, significantly suppresses tumor growth and prolongs survival compared to either agent alone, without inducing systemic toxicity. The observed reduction in Ki-67 and increase in cleaved caspase-3 further support a shift toward reduced proliferation and increased apoptosis in response to treatment. These results validate our hypothesis that targeting F-actin dynamics represents a viable strategy to sensitize DTP cells to conventional chemotherapy. Importantly, this approach directly disrupts the structural plasticity and survival mechanisms that define the DTP phenotype, thereby preventing tumor adaptation and relapse. Taken together, the combination of F-actin polymerization inhibitors with standard chemotherapeutics may offer a promising avenue for addressing drug resistance in HNSCC and potentially other solid tumors characterized by persistent subclonal populations.

**Fig. 7:**
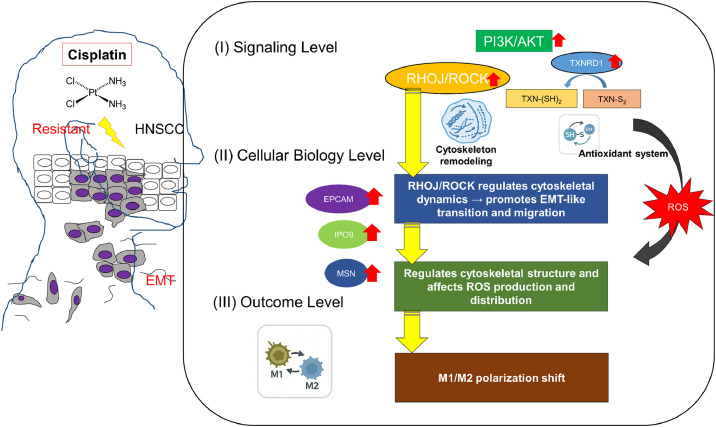
Proposed Model of RHOJ-Mediated Chemoresistance and Immune Modulation in Cisplatin-Resistant HNSCC. RHOJ is upregulated in HNSCC DTP cells and takes effect through the IPO9/EpCAM and rho kinase signaling axes. RHOJ regulates cytoskeletal structure and controls the intracellular distribution of reactive oxygen species, thereby promoting redox homeostasis, reducing ferroptosis, and sustaining cell survival under cisplatin stress. RHOJ also activates TECs, supports M2 macrophage polarization, and suppresses M1 antitumor immunity. Upregulation of TXNRD1 and SOD2 contributes to antioxidant capacity, and PI3K/AKT signaling activation further supports tumor survival. Inhibition of RHOJ signaling may disrupt this adaptive network, sensitizing tumors to chemotherapy and restoring immune responsiveness.

As shown in Table 1, our clinical cohort reflects the epidemiologic distribution in Taiwan, where HPV-positive oropharyngeal cancers account for only 25–30 % of cases; thus, the majority of available specimens were nonviral HNSCC. Within this context, our tissue analyses demonstrated that RHOJ expression was significantly elevated in recurrent, cisplatin-resistant tumors and correlated with pAKT activation, implicating RHOJ in AKT-mediated stress-adaptation signaling. Spatial analyses further revealed RHOJ localization in tumor cells, TECs, and CD206⁺ M2 macrophages, linking RHOJ to angiogenesis and immunosuppressive remodeling. Transcriptomic correlations with RAC1, STAT6, and SOD2, together with protein-level associations involving TXNRD1 and pAKT, support a model in which RHOJ orchestrates cytoskeletal plasticity, redox adaptation, and chemoresistant phenotypes specifically within HPV-negative HNSCC. Its dual promotion of EMT and immune suppression makes RHOJ a particularly attractive therapeutic target. The regulation of RHOJ expression by IPO9/EpCAM further highlights opportunities for multitargeted disruption of the feedback circuits sustaining drug tolerance. Although latrunculin B effectively disrupts F-actin polymerization and serves as a useful mechanistic probe, its limited clinical experience, formulation challenges, and potential systemic toxicity preclude immediate translational application. Our expanded survival experiments (*n* = 15 per group) highlight that small sample sizes can obscure treatment-related adverse effects, underscoring the need for rigorous preclinical evaluation. Future translational development of RHOJ–cytoskeletal–targeting strategies will require dose-escalation studies, pharmacokinetic and biodistribution analyses, toxicology assessments, and exploration of more selective and drug-like cytoskeletal modulators. These steps are essential to ensure safety, optimize therapeutic windows, and determine whether targeting the RHOJ axis can be feasibly integrated into combination regimens for chemoresistant HNSCC. Therefore, the translational implication lies not in advancing Latrunculin B, but in identifying the RHOJ–cytoskeletal axis as a druggable vulnerability, which may guide the future development of more selective and pharmacologically suitable inhibitors (Rho GTPase modulators or ROCK-pathway–targeting compounds). The concept of combining chemotherapy with agents that target this axis represents a promising strategy to eliminate DTP cells and prevent relapse. Future studies should delineate the temporal dynamics of RHOJ activation during chemotherapy exposure and investigate its interactions with additional resistance-associated pathways, such as YAP/TAZ signaling or hypoxia-driven programs. Furthermore, evaluating the clinical significance of RHOJ expression in larger patient-derived cohorts and determining its association with therapeutic outcomes or metastatic progression will be critical. Such efforts may ultimately enable biomarker-driven clinical trials that assess the utility of targeting the RHOJ axis. Overall, our results position RHOJ as a central node linking EMT, cytoskeletal regulation, endothelial remodeling, and immune modulation in HNSCC DTP cells, and support the development of interventions that target this pathway to sensitize tumors, restore immune surveillance, and impede metastatic dissemination. The schematic abstract in Fig. 7 illustrates the therapeutic significance of disrupting cytoskeletal dynamics in drug-tolerant persister (DTP) cells within a head-and-neck squamous cell carcinoma (HNSCC) model.

## Conclusion

Our data identify RHOJ as a pivotal driver of chemoresistance in HNSCC. RHOJ induces epithelial–mesenchymal transition (EMT), enhances cellular motility, and sustains survival under cisplatin-induced stress through the IPO9/EpCAM and Rho-kinase signaling axes. Elevated RHOJ remodels the actin cytoskeleton and modulates intracellular reactive oxygen species distribution, thereby maintaining redox balance, limiting ferroptosis, and supporting tumor cell viability. In parallel, RHOJ activates tumor-associated endothelial cells, promotes M2 macrophage polarization, and suppresses M1-driven antitumor immunity. Up-regulation of TXNRD1 and SOD2, together with PI3K/AKT activation, further enhances antioxidant capacity and stress-adaptive signaling. Pharmacologic disruption of RHOJ-dependent cytoskeletal dynamics using the mechanistic probe Latrunculin B re-sensitizes drug-tolerant persister cells to chemotherapy, highlighting the therapeutic vulnerability of this axis. Although Latrunculin B itself is not clinically viable, our findings support the development of more selective inhibitors targeting the RHOJ/Rho-kinase pathway. Such interventions—alone or combined with standard chemotherapy—may help eliminate persistent drug-tolerant populations, suppress tumor progression, and restore antitumor immune responses.

## Abbreviations

Head and Neck Squamous Cell Carcinoma (HNSCC); Drug-Tolerant Persister (DTP); Epithelial–Mesenchymal Transition (EMT); Ras Homolog Family Member J (RHOJ); Importin 9 (IPO9);

Epithelial Cell Adhesion Molecule (EpCAM); Tumor Microenvironment (TME); Tumor-Associated Macrophage (TAM); Classically (M1) and Alternatively (M2) Activated Macrophages; Short Hairpin RNA (shRNA)

## Institutional review board statement

This study was conducted in accordance with the ethical principles outlined in the Declaration of Helsinki and the Human Subjects Research Act in Taiwan. All procedures involving human participants were reviewed and approved by the Institutional Review Board of Chang Gung Memorial Hospital (IRB No. 202301566A3 & IRB No. 202402023B0). Formalin-fixed, paraffin-embedded tumor specimens were retrospectively obtained from 60 patients with cisplatin-resistant head and neck squamous cell carcinoma (HNSCC) who underwent surgical resection at Chang Gung Memorial Hospital between January 2015 and July 2020. Prior to tissue collection, written informed consent was obtained from all participants. All clinical and pathological data were handled with strict confidentiality. Archived samples were accessed through the CGMH Biobank, and all data analyses were performed in accordance with approved protocols.

## Informed consent statement

Written informed consent was obtained from all patients prior to surgical resection and tissue specimen collection. Participants were fully informed about the purpose of the study, the procedures involved, and their rights to withdraw at any time without affecting their medical care. All patient data and clinical information were de-identified and handled with strict confidentiality in accordance with the ethical standards approved by the Institutional Review Board of Chang Gung Memorial Hospital (IRB No. 202301566A3 & IRB No. 202402023B0).

## Data availability statement

Detailed experimental procedures, characterization of newly synthesized compounds, and additional supporting data are included in the Supplementary Materials. All authors have reviewed and approved the final version of the manuscript, affirming their consent and responsibility for the accuracy and integrity of the work. The datasets used and analyzed during this study are available from the corresponding author upon reasonable request.

## Funding statement

This study was supported by the National Science and Technology Council (NSTC) of Taiwan, with research grants awarded to Jo-Ting Tsai (MOST 111–2314-B-038–073-MY3), Chi-Tai Yeh (MOST 111–2314-B-038–139; NSTC 113–2314-B-038–125), and Hang Huong Ling (NSTC 113–2314-B-182A-018; CMRPG2N0442).

## CRediT authorship contribution statement

**Hang Huong Ling:** Investigation, Funding acquisition, Formal analysis, Data curation, Conceptualization. **Chih-Ming Huang:** Investigation, Funding acquisition, Formal analysis, Data curation, Conceptualization. **Ming-Shou Hsieh:** Investigation, Funding acquisition, Formal analysis, Data curation, Conceptualization. **Vijesh Kumar Yadav:** Investigation, Funding acquisition, Formal analysis, Data curation, Conceptualization. **Iat-Hang Fong:** Investigation, Funding acquisition, Formal analysis, Data curation, Conceptualization. **Kuang-Tai Kuo:** Investigation, Funding acquisition, Formal analysis, Data curation, Conceptualization. **Chi-Tai Yeh:** Writing – review & editing, Writing – original draft, Visualization, Validation, Supervision, Software, Resources, Project administration. **Jo-Ting Tsai:** Writing – review & editing, Writing – original draft, Visualization, Validation, Supervision, Software, Resources.

## Declaration of competing interest

The authors declare the following financial interests/personal relationships which may be considered as potential competing interests: Prof. Chi-Tai Yeh reports a relationship with Taipei Medical University Shuang Ho Hospital Ministry of Health and Welfare that includes: funding grants and non-financial support. Prof. Chi-Tai Yeh has patent issued to Assignee. None If there are other authors, they declare that they have no known competing financial interests or personal relationships that could have appeared to influence the work reported in this paper.

## Appendix Supplementary materials

Supplementary Table S1. Q-PCR primer list. Supplementary Figure S1. Flowchart illustrating patient screening, eligibility criteria, exclusion steps, and final inclusion of HNSCC cases for IHC and mechanistic analyses. The diagram outlines the derivation of cisplatin-sensitive and cisplatin-resistant subgroups and the integration of clinical stratification variables listed in Table 1. Supplementary Figure S2. Validation of RHOJ-dependent phenotypes in additional HNSCC cell lines. Key mechanistic assays were repeated in SAS and FaDu cells to confirm the generalizability of the findings. Image, application 1
