# Supplementary material for: RHOJ-induced chemotherapy resistance through epithelial–mesenchymal transition in drug-tolerant persister cells of head and neck cancer
Source: Transl Oncol. 2026 Jan 17;65:102673. doi: 10.1016/j.tranon.2026.102673 (PMC12856175; doi:10.1016/j.tranon.2026.102673)
Supplement: Supplementary file 1 — Supplementary Table S1. Q-PCR primer list. Supplementary Figure S1. Flowchart illustrating patient screening, eligibility criteria, exclusion steps, and final inclusion of HNSCC cases for IHC and mechanistic analyses. The diagram outlines the derivation of cisplatin-sensitive and cisplatin-resistant subgroups and the integration of clinical stratification variables listed in Table 1. Supplementary Figure S2. Validation of RHOJ-dependent phenotypes in additional HNSCC cell lines. Key mechanistic assays were repeated in SAS and FaDu cells to confirm the generalizability of the findings. [file mmc1.docx]

**SUPPLEMENTARY DATA**

RHOJ-induced chemotherapy resistance through epithelial–mesenchymal transition in drug-tolerant persister cells of head and neck cancer

Hang Huong Ling^1,2^, Chih-Ming Huang^3,4^, Ming-Shou Hsieh^5^, Vijesh Kumar Yadav^5^, Iat-Hang Fong^5^, Kuang-Tai Kuo^6,7^, Chi-Tai Yeh^5^*, Jo-Ting Tsai ^1,8,9^*

^1^ Graduate Institute of Clinical Medicine, College of Medicine, Taipei Medical University, Taipei City, 110, Taiwan.

^2^ Division of Hemato-oncology, Department of Internal Medicine, Chang Gung Memorial Hospital, Keelung and Chang Gung University, College of Medicine, Keelung, 204, Taiwan.

^3^ Department of Otolaryngology, Taitung Mackay Memorial Hospital, Taitung City 950408, Taiwan;

^4^ Department of Nursing, Tajen University, Pingtung 90741, Taiwan

^5^ Division of Hematology and Oncology, Taipei Medical University Shuang Ho Hospital, New Taipei City, 23561, Taiwan

^6^ Division of Thoracic Surgery, Department of Surgery, School of Medicine, College of Medicine, Taipei Medical University, Taipei 110, Taiwan.

^7^ Division of Thoracic Surgery, Department of Surgery, Taipei Medical University-Shuang Ho Hospital, New Taipei City 235, Taiwan.

^8^Department of Radiology, School of Medicine, College of Medicine, Taipei Medical University, Taipei City, 110, Taiwan.

^9^Department of Radiation Oncology, Cancer Center, Taipei Medical University—Shuang Ho Hospital, New Taipei City 23561, Taiwan.

*Authors to whom correspondence should be addressed.

Prof. Chi-Tai Yeh, PhD. Department of Medical Research & Education, Taipei Medical University - Shuang Ho Hospital, New Taipei City, 235, Taiwan. Phone: 886-2-2490088 ext. 8885; FAX: 886-2-2248-0900. E-mail: [ctyeh@s.tmu.edu.tw](mailto:ctyeh@s.tmu.edu.tw)

Prof. Jo-Ting Tsai, MD, PhD, Department of Radiation Oncology, Cancer Center, Taipei Medical University—Shuang Ho Hospital, New Taipei City 23561, Taiwan; Phone: 886-2-2490088 ext. 8881; FAX: 886-2-2248-0900. E-mail: [10576@s.tmu.edu.tw](mailto:10576@s.tmu.edu.tw)

**Supplementary Table S1.** Q-PCR primer list.

| Primer | forward | reverse |
| --- | --- | --- |
| RHOJ | TTGCTCGGACTGTATGACACCG | CCTGGACATTGTGGTAAGAGGC |
| IPO9 | GTCATCCTTGCAGACCTCAACC | GTTGCCTGTAGGAACTGCTGGA |
| MSN | CTGATGGAGAGGCTGAAGCAGA | ACGCTTCCGTTCCTGCTCAAGT |
| EPCAM | GCCAGTGTACTTCAGTTGGTGC | CCCTTCAGGTTTTGCTCTTCTCC |
| TGFB1 | TACCTGAACCCGTGTTGCTCTC | GTTGCTGAGGTATCGCCAGGAA |
| CTNNB1 | CACAAGCAGAGTGCTGAAGGTG | GATTCCTGAGAGTCCAAAGACAG |

s
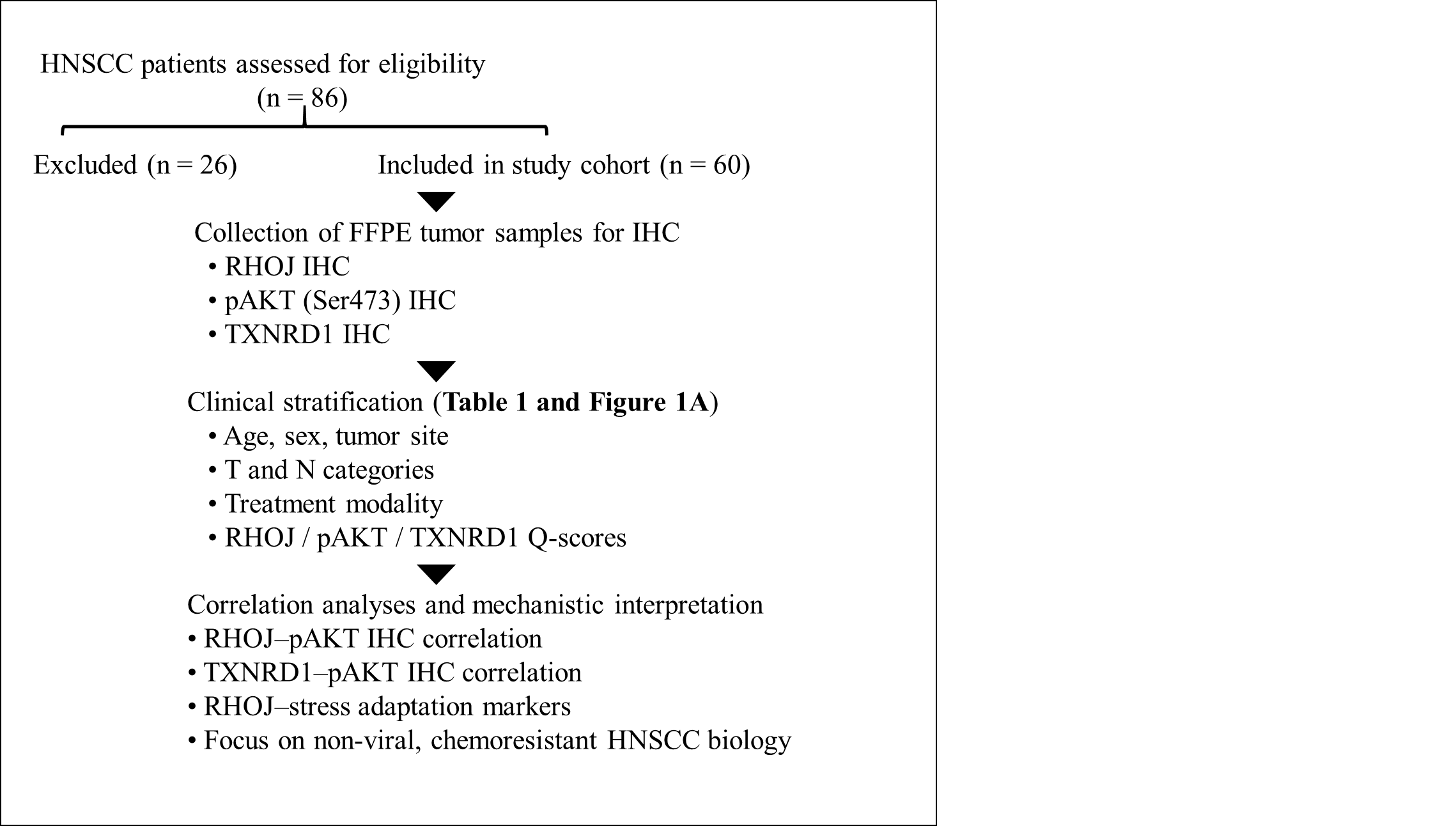


**Supplementary Figure S1.** Flowchart illustrating patient screening, eligibility criteria, exclusion steps, and final inclusion of HNSCC cases for IHC and mechanistic analyses. The diagram outlines the derivation of cisplatin-sensitive and cisplatin-resistant subgroups and the integration of clinical stratification variables listed in **Table 1.**


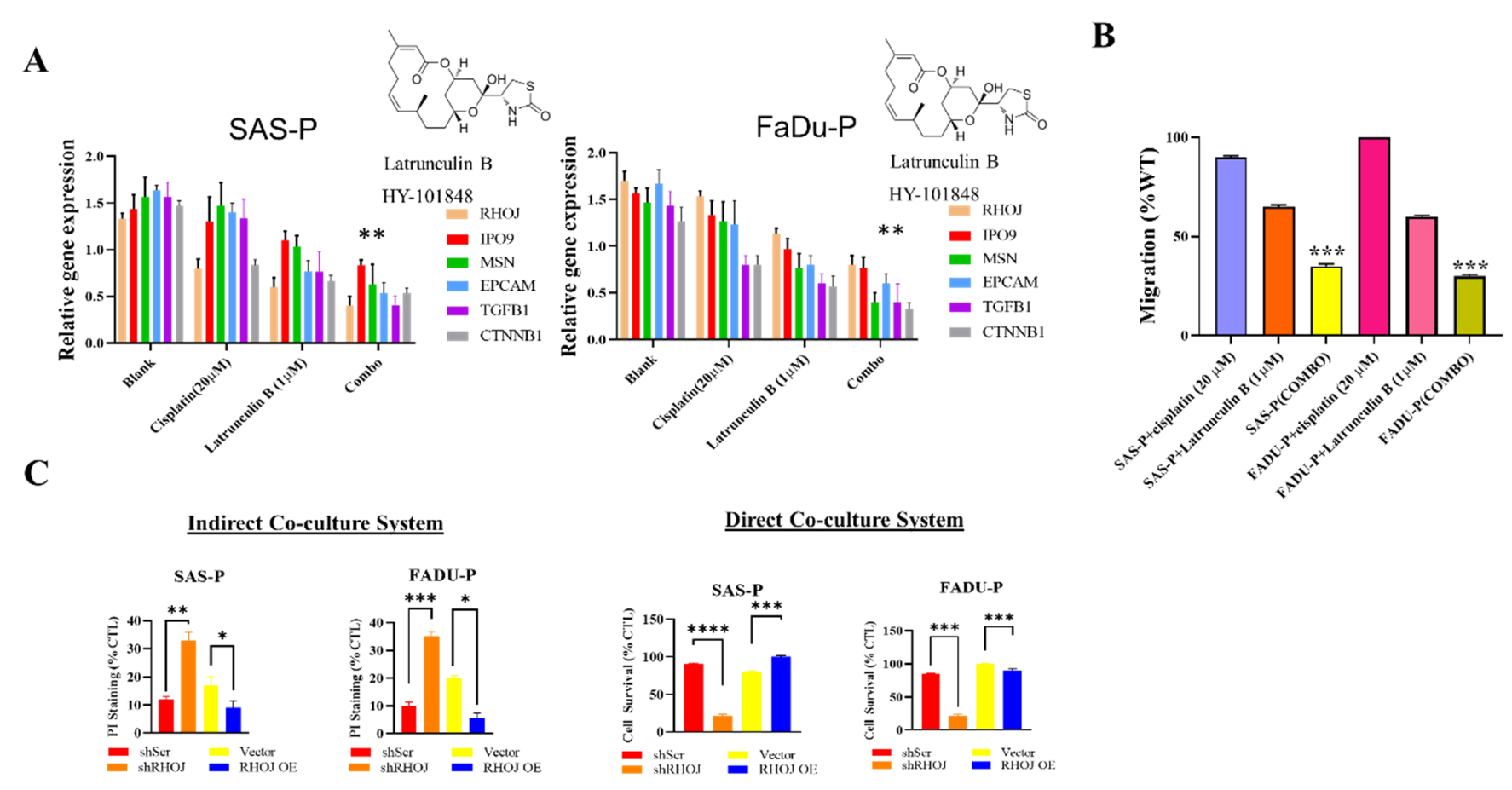


**Supplementary Figure S2.** Validation of RHOJ-dependent phenotypes in additional HNSCC cell lines. Key mechanistic assays were repeated in SAS and FaDu cells to confirm the generalizability of the findings.
